# Data describing the effect of *DRD4* promoter polymorphisms on promoter activity

**DOI:** 10.1016/j.dib.2016.03.084

**Published:** 2016-04-01

**Authors:** Shoin Tei, Hiroaki Mitsuhashi, Shoichi Ishiura

**Affiliations:** Department of Life Sciences, Graduate School of Arts and Sciences, The University of Tokyo, 3-8-1 Komaba, Meguro-ku, Tokyo, Japan

## Abstract

This data article tested whether polymorphisms within the dopamine D4 receptor (*DRD4*) gene promoter can lead to differences in the promoter activity. The variants, a 120-bp variable number tandem repeat (VNTR), −906 T/C, −809 G/A, −616G/C, and −521C/T, were introduced into the *DRD4* promoter and the promoter activity was measured in a neural cell line using the luciferase assay. However, no differences were detected among the haplotypes investigated, and the *in vitro* data obtained from our protocol could not support the involvement of *DRD4* promoter polymorphisms in heritable human traits.

## **Specification Table**

**Table:** 

Subject area	*Biology*
More specific subject area	*Molecular biology, Genetics*
Type of data	*Table, image, graph*
How data was acquired	*RT-PCR, Luciferase assay*
Data format	*Raw, analysed*
Experimental factors	*Polymorphisms (120-bp VNTR, rs3758653 for −906 T/C, rs936461 for −809 G/A, rs747302 for −616 G/C, and rs1800955 for −521 C/T) were introduced into the promoter sequence of the DRD4 gene*
Experimental features	*DRD4 expression was detected by RT-PCR using cDNA from SH-SY5Y cells. Firefly luciferase gene downstream of DRD4 promoter was expressed in SH-SY5Y cells, and the luciferase activity of each construct was measured 48 h after transfection*
Data source location	*University of Tokyo, Japan*
Data accessibility	*Data supplied with this article*

## Value of the data

• We examined the effect of *DRD4* promoter polymorphisms on gene expression in an *in vitro* reporter gene experiment.
• This data is useful for characterising the link between heritable mental traits and the polymorphisms.
• Our data can provide insight into methodology and considerations for investigation of polymorphisms in non-coding regions.

## 1 Data

**Fig. 1:**
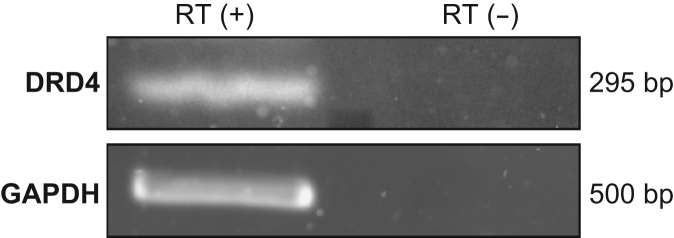
RT-PCR analysis of *DRD4* gene expression. *DRD4* expression in SH-SY5Y cells was detected using RT-PCR. The housekeeping gene *GAPDH* was amplified as an internal control.

Endogenous dopamine D4 receptor (*DRD4*) gene expression in SH-SY5Y cells was detected by RT-PCR using cDNA derived from the cell line (Fig. 1).

**Fig. 2:**
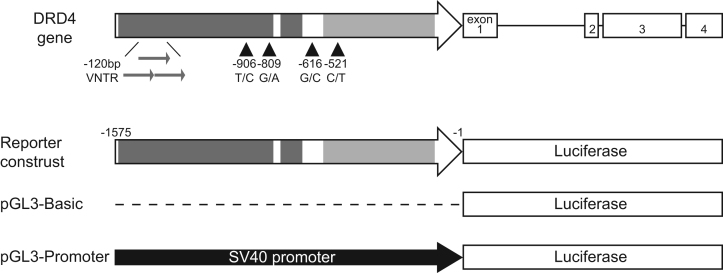
Schematic representation of the *DRD4* gene and the reporter construct with the locations of the polymorphisms studied. The DNA fragment cloned here corresponded to region −1576 to −1 relative to the translation start site, and was the longest among functional assays on mutated DRD4 promoter;−591 to −123 was cloned in Okuyama et al [2], −1389 to −1203 in D׳Souza et al. [3], −668 to −389 in Kreszturi et al. [4] and −1571 to −389 in Kreszturi et al. [5].The putative silencer (dark grey boxes, −1571 to −800 and −770 to −678) and enhancer (light grey box, −591 to −123) regions are indicated in the *DRD4* gene promoter (white arrow) [1], [4]. The 120-bp VNTR is 1.2 kb upstream from the initial codon, and −521C/T is a C/T SNP at −521 in the promoter region (the description can be applied to the other SNPs). The promoter was cloned upstream of the firefly luciferase gene, so that luciferase expression was driven by the promoter. pGL3 promoter which contains SV40 promoter upstream luciferase gene was used as positive control.

**Fig. 3:**
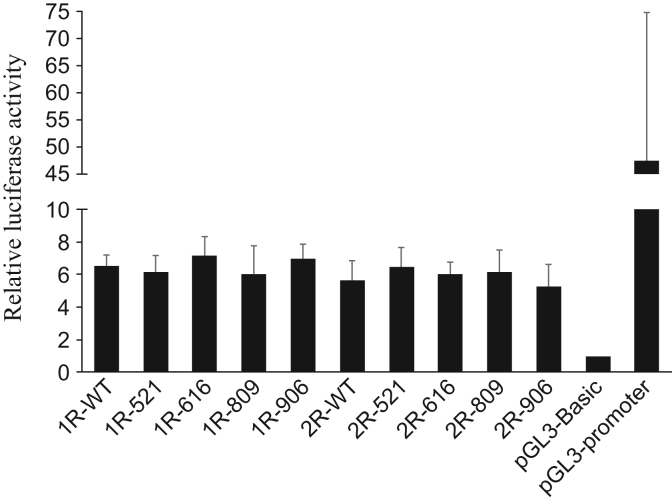
The effect of the polymorphisms on the *DRD4* promoter activity. *DRD4* promoter activity was measured as the luciferase activity in SH-SY5Y cells. The relative luciferase activity of pGL3-Basic was defined as 1 and pGL3 promoter was used as positive control. The assay failed to detect any significant differences between haplotypes. Data are expressed as means±SD (*n*=5) (Tukey–Kramer test, **p<0.01).

**Fig. 4:**
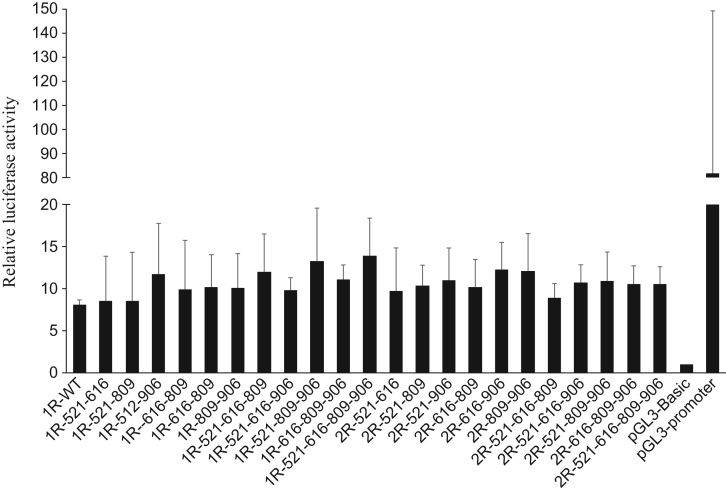
The effect of the combined polymorphisms on the *DRD4* promoter activity. *DRD4* promoter activity was measured as luciferase activity in SH-SY5Y cells. The relative luciferase activity of pGL3-Basic was defined as 1 and pGL3 promoter was used as positive control. The assay failed to detect any significant differences between haplotypes. Data are expressed as means±SD (*n*=4) (Tukey–Kramer test, **p<0.01).

To test whether the polymorphisms within the promoter change the promoter activity, luciferase activity was measured under the influence of the *DRD4* promoter into which polymorphisms were introduced (Fig. 2 and Table 1). All of the reporter plasmids containing the *DRD4* fragment exhibited significantly higher luciferase activity than the control pGL3-Basic, and although every possible combination of haplotypes was investigated, there were no activity differences among the introduced mutations in SH-SY5Y cells (Fig. 3, Fig. 4).

## 2 Experimental design, materials and methods

### 2.1 Construction of reporter plasmid

A DNA fragment spanning −1576 to −1 of the *DRD4* promoter region was amplified from human genomic DNA with TaKaRa LA *Taq* (TaKaRa) and inserted into pCR-Blunt (Life Technology). The cloned sequence was confirmed by Sanger sequencing and shown in Supplementary Fig. 1. Mutations were introduced using PCR-based site-directed mutagenesis for the four SNPs, and with *Not*I treatment for the VNTR; DNA ligation after *Not*I treatment converts a 2-repeat allele into a 1-repeat allele because one *Not*I recognition site is present within the repeat. The mutated insertion was subcloned into the *Xho*I and *Hin*dIII sites of pGL3-Promoter Vector (Promega) replacing the original SV40 promoter with the *DRD4* promoter. The primer sequences used for construction are shown in Table 2.

### 2.2 Cell culture and transfection

Human neuroblastoma SH-SY5Y cells were cultured in Dulbecco׳s Modified Eagle׳s Medium (Sigma-Aldrich) supplemented with 10% foetal bovine serum (Invitrogen) at 37 °C in a humidified atmosphere containing 5% CO_2_. Twenty-four hours before transfection, cells were plated at 4×10^5^ cells/well in 96-well plates.

The reporter plasmid (0.2 ng/well) and pRL-TK (0.01 ng/well) were transfected into SH-SY5Y cells with 0.06 μL/well FuGENE 6 Transfection Reagent (Promega), according to the manufacturer׳s protocol.

### 2.3 Luciferase assay

Forty-eight hours after transfection, the luciferase activity was measured in quadruplicate with the Dual-Glo Luciferase assay System (Promega) using Centro LB960 (Berthold), following the manufacturer׳s instructions. Relative luciferase activity was calculated as the ratio of firefly to *Renilla* luciferase activity.

### 2.4 Total RNA isolation and RT-PCR

Total RNA of SH-SY5Y was extracted with GenElute Mammalian Total RNA Miniprep Kit (Sigma-Aldrich). First-strand cDNA was synthesised from extracted RNA using Prime Script RT Reagent Kit with gDNA Eraser (Perfect Real Time) (TaKaRa). *DRD4* mRNA expression was detected using TaKaRa LA *Taq*, as described [1]. To verify the procedure, glyceraldehyde-3-phosphate dehydrogenase (GAPDH) was amplified as an internal control. The primer sequences used for RT-PCR are shown in Table 2.

## Figures and Tables

**Table 1:** The constructed haplotypes consisted of 120-bp VNTR and four SNPs of the *DRD4* gene.

Reporter construct	120 bp VNTR	−906 C/T	−809 G/A	−616 G/C	−521 C/T
1R-WT	1	T	G	G	C
1R-521	1	T	G	G	**T**
1R-616	1	T	G	**C**	C
1R-809	1	T	**A**	G	C
1R-906	1	**C**	G	G	C
2R-WT	2	T	G	G	C
2R-521	2	T	G	G	**T**
2R-616	2	T	G	**C**	C
2R-809	2	T	**A**	G	C
2R-906	2	**C**	G	G	C
1R-521-616	1	T	G	**C**	**T**
1R-521-809	1	T	**A**	G	**T**
1R-521-906	1	**C**	G	G	**T**
1R-616-809	1	T	**A**	**C**	C
1R-616-906	1	**C**	G	**C**	C
1R-809-906	1	**C**	**A**	G	C
1R-521-616-809	1	T	**A**	**C**	**T**
1R-521-616-906	1	**C**	G	**C**	**T**
1R-521-809-906	1	**C**	**A**	G	**T**
1R-616-809-906	1	**C**	**A**	**C**	C
1R-521-616-809-906	1	**C**	**A**	**C**	**T**
2R-521-616	2	T	G	**C**	**T**
2R-521-809	2	T	**A**	G	**T**
2R-521-906	2	**C**	G	G	**T**
2R-616-809	2	T	**A**	**C**	C
2R-616-906	2	**C**	G	**C**	C
2R-809-906	2	**C**	**A**	G	C
2R-521-616-809	2	T	**A**	**C**	**T**
2R-521-616-906	2	**C**	G	**C**	**T**
2R-521-809-906	2	**C**	**A**	G	**T**
2R-616-809-906	2	**C**	**A**	**C**	C
2R-521-616-809-906	2	**C**	**A**	**C**	**T**

**Table 2:** Primer sequences and application.

Application	Forward	Reverse
Amplification of DRD4 promoter	ACCActcgaGAGGCTGGGCTGGACTCGCCGTTT	AAGGaagcttGGCGCGCCCGGGCGG
	^⁎^The lower-case letters represent XhoI or HindIII restriction sites.	
Nucleotide substitution −916 T>C	GAAGAGTCCATAGAACTCTCTGCTGCGCTTTGC	GCAAAGCGCAGCAGAGAGTTCTATGGACTCTTC
Nucleotide substitution −809 G>A	CGAGCCGAACCTACTGTCCGGTCCCG	CGGGACCGGACAGTAGGTTCGGCTCG
Nucleotide substitution −616 G>C	GCGGGGGCTGAGCACCAGAGGCTGC	GCAGCCTCTGGTGCTCAGCCCCCGC
Nucleotide substitution −521 T>C	GCGTGGAGGGCGCGCACGAGG	CCTCGTGCGCGCCCTCCACGC
	^⁎^The underlined letters correspond to each SNP	
RT-PCR of *DRD4*	GCACCGCCTCCATCTTCAACC	CGGAACGTGGCCCAGTAGAGC
RT-PCR of *GAPDH*	AAGGCTGAGAACGGGAAGCTTGTCATCAAT	TTCCCGTCTAGCTCAGGGATGACCTTGCCC

## Footnotes

Appendix A Supplementary data associated with this article can be found in the online version at doi:10.1016/j.dib.2016.03.084.

## References

[1] Kamakura S., Iwaki A., Matsumoto M., Fukumaki Y. (1997). Cloning and characterization of the 5′-flanking region of the human dopamine D4 receptor gene. Biochem. Biophys. Res. Commun..

[2] Okuyama Y., Ishiguro H., Nankai M., Shibuya H., Watanabe A., Arinami T. (2000). Identification of a polymorphism in the promoter region of DRD4 associated with the human novelty seeking personality trait. Mol. Psychiatry.

[3] D׳Souza U.M., Russ C., Tahir E., Mill J., McGuffin P., Asherson P.J., Craig I.W. (2004). Functional effects of a tandem duplication polymorphism in the 5′flanking region of the DRD4 gene. Biol. Psychiatry.

[4] Kereszturi E., Kiraly O., Barta C., Molnar N., Sasvari-Szekely M., Csapo Z. (2006). No direct effect of the −521 C/T polymorphism in the human dopamine D4 receptor gene promoter on transcriptional activity. BMC Mol. Biol..

[5] Kereszturi E., Kiraly O., Csapo Z., Tarnok Z., Gadoros J., Sasvari-Szekely M., Nemoda Z. (2007). Association between the 120-bp duplication of the dopamine D4 receptor gene and attention deficit hyperactivity disorder: genetic and molecular analyses. Am. J. Med. Genet. B: Neuropsychiatr. Genet..

