# Supplementary material for: Data describing the effect of DRD4 promoter polymorphisms on promoter activity
Source: Data Brief. 2016 Apr 1;7:1112–7. doi: 10.1016/j.dib.2016.03.084 (PMC4833125; doi:10.1016/j.dib.2016.03.084)
Supplement: Supplementary file 1 — Supplementary material [file mmc1.pdf]

GAGGCTGGGCTGGACTCGCCGTTTGGCGCCCAGCCGGAGACCCCTGGGCCACCCTC
CCCGGTTCCATCCTGGGAGAGAAGAAACTTCCACTGTCCCAGTCTCTCCTGTGGTGA
CTTCTTTTGCTCAAGGGTTCACAAGGGCAGCAGACTGATCTGCACAAGAGGGACTG
AGCCTGGCTGCGGCCGCACCTGATGCCTGTGCACACCTGTCCCTGGTG GTCTCTCC
TGAGGTGACTTCTTTTGCTCAAGGGTTCACAAGGGCAGCAGACTGATCTGCACAAG
AGGGACTGAGCCTGGCTGCGGCCGCACCTGATGCCTGTGCACACCTGTCCCTGGTG
CAGGCCCTTCCCCCGCAGCCCCGGTGACCTTGTCCTGTGAGGCTCACGGTGCCTGCT
CCTTCTCAGAGTCCTCATTTCTCCCTTTAACGCACGCCCTGTCAGCGCCTCCGTCTAG
CCCGTGTCCTCCAGACCTTTGAGGTCAGGGGCCTCAATTCCTGGCACAGCCCCTCCA
CTCCAGGCCTCCCAGAAACAGACTAGAGCATCAGGATGTGCGCCGCCTGGGGTCCC
ACAGAGTGGTGCCCCCTTTTAGTGTCTTCTAGGCCCCTTAGTGACAGACTACAGAAA
ATACCTCTCAGGTCACAGGTCACCCCTCTTTGGTGAAGAGTCCATAGAATTCTCTGC
TGCGCTTTGCAAGCACTTTCTCTTCTGCACGTTTGGAACCTACCCCGGCCTGTCGTGT
CTTTCTCCTGGCCTCCTCGCGAGCCGAACCTGCTGTCCGGTCCCGGGACCCCCTGCC
CAGGGTCAGAGGGGCGCCTACCTAGCTCACGGTCTTGGGCCGGAGGGAATGGAGGA
GGGAGCGGGGTCGACCGCTCAGCTGTCCGCCCAGTTTCGGAGGCGGCCACGCGAG
GATCAACTGTGCAACGGGTGGGGCCGCGGCTGACCGTGGTGGTCGCGGGGGCTGAG
GACCAGAGGCTGCGGGGGGGGGCGGCGGGATGAGCTAGGCGTCGGCGGTTGAGTC
GGGCGCGGAGTCGGGGGCAGGGGGAGCGGGCGTGGAGGGTGCGCACGAGGTCGA
GGCGAGTCCGCGGGGGAGGCGGGCAGAGCCTGAGCTCAGGTCTTTCTGCGTCTGGC
GGAACGGGCCTGGGAGGGAGGTTTTGCCAGATACCAGGTGGACTAGGGTGAGCGCC
CGAGGGCCGGGACGCACGCACGGGCCGGGTAGGATGGCGCTGGCGTCGATGCCCGC
GCGCTTCAGGGCCTGGTCTGGCCGCCCCTCCATCCTTGTCGGTTTCTCGGGTCGCGG
ACCCCGCGCGGCGCCGGGCGATGCTGGCCTGCCCGTGGCCACCACCTCGCTTCATTC
CCGTCTCTTTGGGCCGCCGCATTCGTCCACGTGCCCGTCTCTCCCTGCGCAAAATTCC
AAGATGAGCAAATACTGGGCTCACGGTGGAGCGCCGCGGGGGCCCCCCTGAGCCGG
GGCGGGTCGGGGGCGGGACCAGGGTCCGGCCGGGGCGTGCCCGAGGGGAGGGACT
CCCCGGCTTGCGACCCGGCGTTGTCCGCGGTGCTCAGCGCCCGCCCGGGCGCGCC

Supplementary Fig. 1. *DRD4* promoter sequence (-1576 to -1) amplified from human genomic DNA and cloned into pGL3-promoter
The 1576-bp sequence contains 120-bp VNTR (boxed) and the four SNPs (underlined)).
